# The role of histone modification and a regulatory single-nucleotide polymorphism (rs2071166) in the Cx43 promoter in patients with TOF

**DOI:** 10.1038/s41598-017-10756-6

**Published:** 2017-09-05

**Authors:** Ruoyi Gu, Jun Xu, Yixiang Lin, Wei Sheng, Duan Ma, Xiaojing Ma, Guoying Huang

**Affiliations:** 1 Children’s Hospital of Fudan University, Shanghai, China; 2 Shanghai Key Laboratory of Birth Defects, Shanghai, China; 3 Key Laboratory of Molecular Medicine, Ministry of Education, Shanghai Medical College, Fudan University, Shanghai, China

## Abstract

Abnormal level of Cx43 expression could result in CHD. Epigenetic modification and disease-associated, non-coding SNPs might influence gene transcription and expression. Our study aimed to determine the role of histone modification and an rSNP (rs2071166) in the Cx43 promoter in patients with TOF. Our results indicate that H3K18ac bind to Cx43 promoter and that their levels are reduced in TOF patients relative to controls. The relationship between the non-coding SNP in the Cx43 gene and TOF patients was evaluated in 158 patients and 300 controls. The C allele of rs2071166 was confirmed to result in an increased risk of TOF (OR = 1.586, 95%CI 1.149–2.189). Individuals with the CC genotype at rs2071166 also showed a significant susceptibility to TOF (OR = 2.961, 95%CI 1.452–6.038). The mRNA level in TOF who were CC genotype was lower than that in patients with the AA/AC genotype. Functional analysis in cells and transgenic zebrafish models showed that rs2071166 decreased the activity of the promoter and could block the interaction between RXRα and RARE. This is the first study to illustrate that epigenetic modification and an rSNP in the Cx43 promoter region play a critical role in TOF by impacting the transcriptional activity and expression level of Cx43.

## Introduction

Congenital heart disease (CHD) is one of the most common birth defects. Genetic factors have been suggested to play an important role in CHD^1^. Tetralogy of Fallot (TOF) is the most common cyanotic CHD, accounting for approximately 10% of all cases of CHD, and includes four cardiac defects: (i) a ventricular septal defect; (ii) right ventricle outflow tract obstruction; (iii) right ventricle hypertrophy and (iv) an over-riding aorta^2^. However, the exact pathophysiology of TOF is not well understood.

Cx43 is the main protein in human myocardial gap junctions^3–5^. It can form gap junction channels between neighboring cells to allow the intercellular exchange of ions and metabolites^5^. Animal studies have shown that abnormal expression of Cx43 results in narrowing of the right ventricular outflow tract, stenosis of the pulmonary artery, and hypertrophy of the right ventricle^6–10^. Some researchers consider TOF to be a neural crest cell-related conotruncal heart malformation that occurs during embryonic development^11^. According to some studies, abnormalities in the expression and distribution of Cx43 in cardiomyocytes may disrupt the migration of cardiac neural crest cells and cause congenital heart diseases, such as TOF^2, 4, 6, 12, 13^. Thus, Cx43 might contribute to the pathogenesis of TOF.

It has been realized that epigenetic modifications (such as histone modification) and non-coding regulatory SNPs (rSNPs) can modulate gene expression and lead to diseases^14–17^. Histone modification includes the post-translational modification of histone protein N-terminal regions by acetylation, methylation, phosphorylation, ubiquitylation, and sumoylation, among others^18^. Among these modifications, histone acetylation is the most widely studied^19^. Evidence has shown that the acetylation of specific lysine residues in the core histone amino tail domains plays a critical role in transcriptional regulation^20^. Thousands of genes and gene variants (such as SNPs) involved in human diseases have been identified in genome-wide association studies (GWASs). SNPs in coding regions can change the amino acids in protein-coding genes and influence protein function, which plays a vital role in disease pathophysiology^21, 22^. Although rSNPs show modest effects that might modulate gene function more subtly than SNPs in coding regions, they can also modulate gene expression through multiple mechanisms including RNA splicing, transcription factor binding, DNA methylation and miRNA recruitment^14, 15^.

We discovered lower acetylation of histone H3 lysine 18 in patients with TOF. The mechanism through which H3K18ac regulates Cx43 transcriptional regulation remains unknown. Our previous study found a functional retinoic acid response element (RARE) in the Cx43 promoter^23^. In the present work, we identified a SNP, rs2071166, located near this functional RARE. However, the function of this SNP is still unclear. Therefore, we conducted several biochemical experiments in the present study to investigate the role of epigenetic modification and this non-coding SNP in Cx43.

## Results

### Histone H3 acetylation at lysine 18 is decreased in the heart tissue of patients with TOF

**Figure 1:**
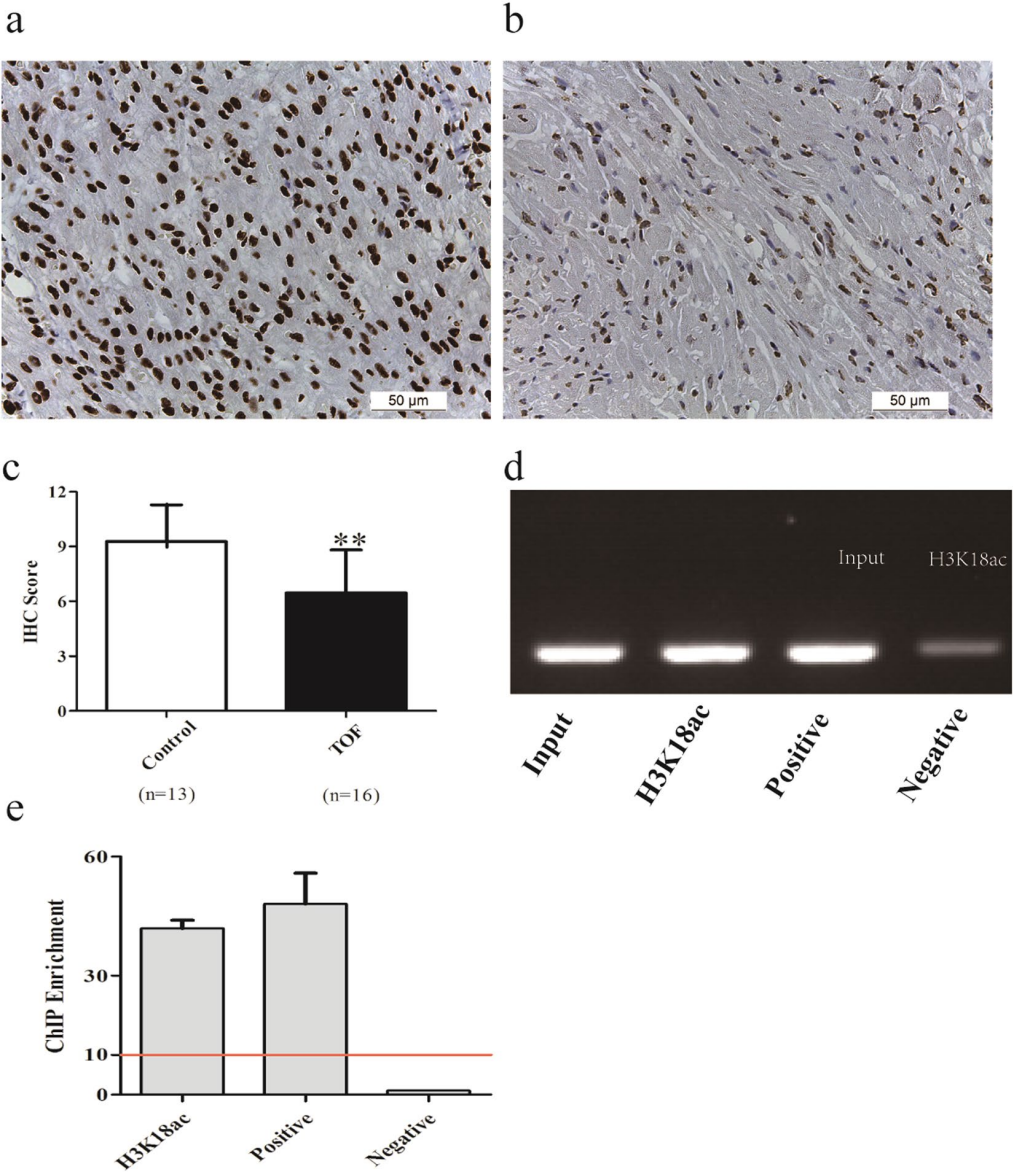
H3K18ac levels are reduced in patients with TOF, and H3K18ac binds to Cx43 directly. (**a**,**b**) Immunohistochemistry (IHC) image (400×) of H3K18ac in control heart tissues (n = 13) and TOF heart tissues (n = 16). (**c**) H3K18ac IHC scores for control and TOF tissues. (**d**,**e**) Chromatin immunoprecipitation assays were performed with an antibody against H3K18ac, IgG (negative control) or RNA Pol II (positive control). Then, 10% input DNA and immunoprecipitated DNA were assayed by PCR and quantitative real-time polymerase chain reaction with Cx43 primers targeting the H3K18ac binding site. *p < 0.05; **p < 0.001. A red line indicates a positive result.

Immunohistochemical (IHC) staining was used to analyze the histone H3 acetylation level at lysine 18 in heart tissue. Staining with an anti-H3K18ac antibody was localized to the nucleus of heart tissues in both the control group and the TOF group. IHC staining in heart tissues from the TOF group was much lower than that in the control group (p < 0.001; Fig. 1a,b and c).

### H3K18ac binding to the Cx43 promoter

We performed a chromatin immunoprecipitation assay (ChIP) to determine whether H3K18ac binds to Cx43. As shown in Fig. 1d, a PCR product corresponding to the Cx43 promoter region was detected when tissues were incubated with anti-acetyl-histone H3 (Lys18). In quantitative real-time PCR, the Cx43 region was enriched by approximately 41.92-fold in the H3K18ac antibody-treated sample relative to the sample treated with normal IgG. Our findings demonstrate that H3K18ac can bind to the Cx43 promoter directly (Fig. 1d and e).

### rs2071166 is associated with an increased risk of TOF

**Figure 2:**
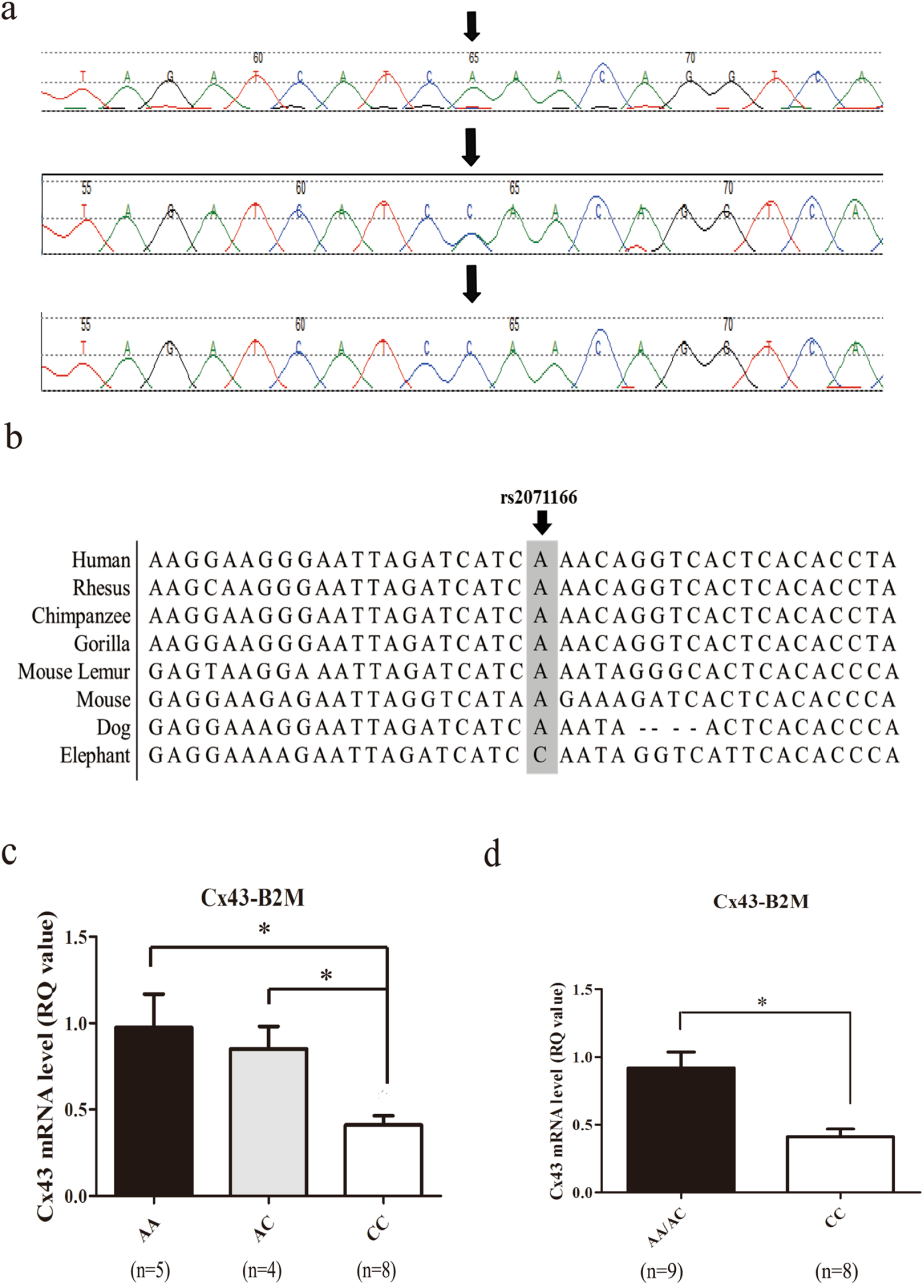
Sequence diagram of the rs2071166 site and the Cx43 mRNA level in patients with different genotypes. (**a**) Arrows indicate the SNP site. Genotype: top, AA; middle, AC; bottom, CC. Green, black, red and blue represent the A allele, G allele, T allele and C allele, respectively. (**b**) The Cx43 promoter region is similar among mammals, such as chimpanzee, gorilla and mouse lemur, etc. The position of the SNP site was marked by black arrows. (**c**,**d**) The relative mRNA expression level of Cx43 was analyzed in samples from 9 patients with the AA/AC genotype and 8 patients with the CC genotype, with B2M as an internal control. Bar, SE. *p < 0.05.

The rs2071166 SNP is located within the functional retinoic acid response element with an MAF > 0.05 in the Chinese Han population in Beijing. In this study, 158 patients with TOF and 300 normal controls were recruited. The genotype frequencies in the control group were evaluated with regard to Hardy-Weinberg equilibrium (HWE). There was no significant departure from HEW (p = 0.204). The association of the SNP genotype and allelotype with the risk of TOF was analyzed with Pearson’s Χ^2^ test. The AA, AC, and CC genotypes were detected at the rs2071166 site in both cohorts (Fig. 2a, Table 1), and the frequency distribution of these three genotypes differed significantly between the control group and the TOF group (p = 0.0068; Table 1). The frequency of the CC genotype was higher in the TOF group (12.6% vs. 4.7%), and the logistic regression analysis revealed that the CC genotype was positively correlated with TOF (p = 0.0019, OR = 2.961, 95% CI 1.452–6.038) (Table 2). The minor allele C of rs2071166 was present at a much higher frequency in the TOF group (26.9% vs. 18.8%), and the logistic regression analysis revealed that the C allele was positively correlated with TOF (p = 0.0048, OR = 1.586, 95% CI 1.149–2.198) (Table 3).

**Table 1:** Association between SNP genotypes.

SNP	Genotype	Frequency		χ^2^	P value
		TOF (%)	Control (%)		
rs2071166	AA	93(58.9)	201(67.0)	9.972	P = 0.0068
	AC	45(28.5)	85(28.3)		
	CC	20(12.6)	14(4.7)

**Table 2:** Association between SNP allelotypes and the risk of TOF.

SNP	Genotype	Frequency		Odds Ratio (95% CI)	χ^2^	P value
		TOF (%)	Control (%)			
rs2071166	AA/AC	138(87.4)	286(95.3)	0.338(0.166–0.689)	9.618	0.0019
	CC	20(12.6)	14(4.7)	2.961(1.452–6.038)

**Table 3:** Association between SNP allelotypes and the risk of TOF.

SNP	Allelotype	Frequency		Odds Ratio (95% CI)	χ^2^	P value
		TOF (%)	Control (%)			
rs2071166	A	231(73.1)	487(81.2)	0.631(0.457–0.870)	7.947	P = 0.0048
	C	85(26.9)	113(18.8)	1.586(1.149–2.189)		

### The homozygous CC genotype exhibits lower transcriptional activity

Studies have shown that DNA polymorphisms in the promoter region can influence gene transcriptional activity^21^. To determine the effect of this polymorphism, qRT-PCR was performed to determine mRNA levels in the tissue of 17 patients with TOF, and the results were analyzed with respect to genotypes with the Mann-Whitney test. The mRNA level of Cx43 in individuals with the CC genotype was approximately 0.44-fold lower than that in individuals with the AA/AC genotype relative to B2M levels (RQ value for each genotype: AA/AC, 0.92 ± 0.12; CC, 0.41 ± 0.06, p < 0.05) (Fig. 2c and d).

### The risk factor ‘C’ allele reduces Cx43 promoter activity

**Figure 3:**
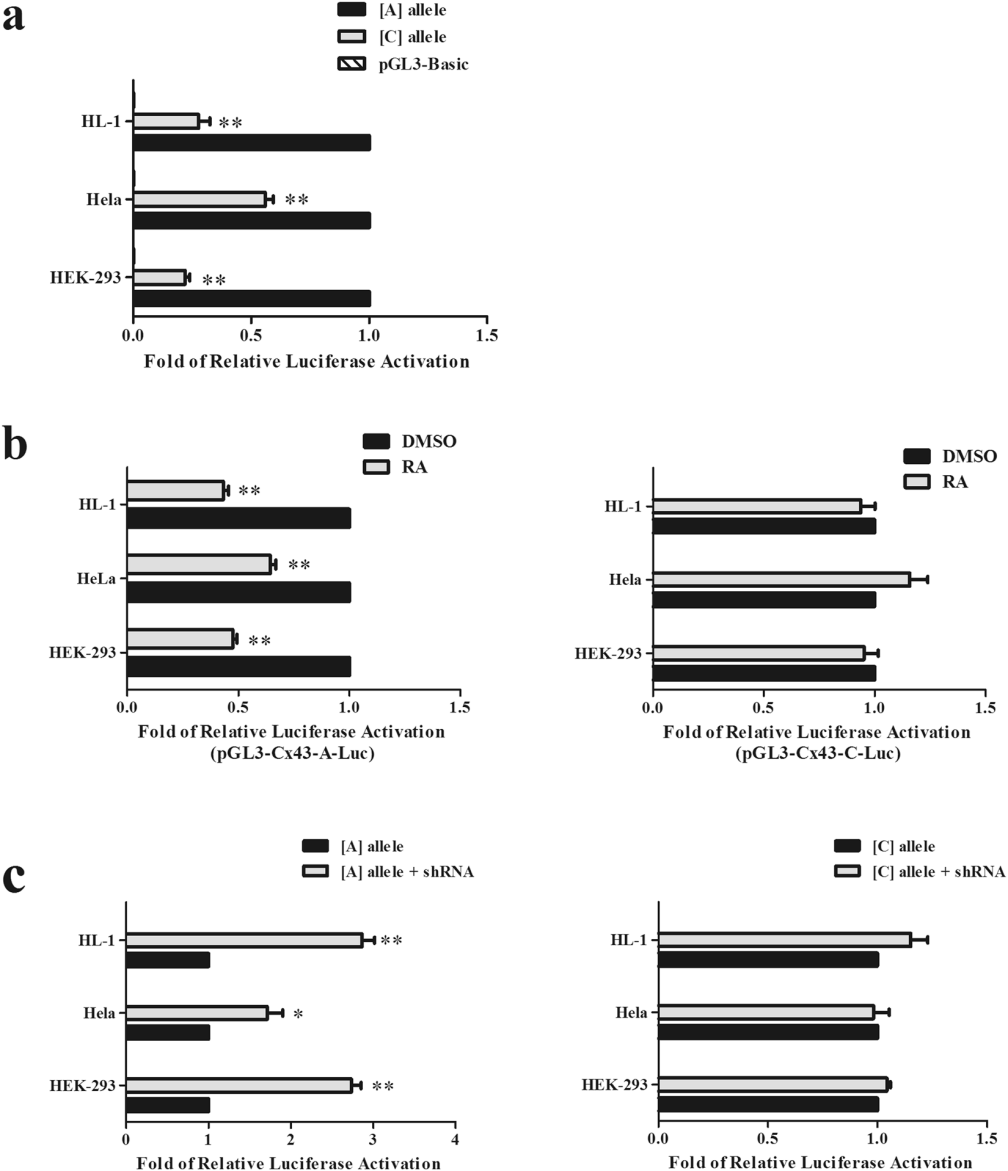
The C allele can influence the function of the RARE in Cx43. (**a**) Luciferase activity analysis of the construct with the A allele and the construct with the C allele. (**b**) The luciferase construct with A allele and the construct with the C allele were co-transfected with human *RXRα* expression vectors into cells, which were then treated with vehicle (DMSO) or ligand (RA). (**c**) Luciferase constructs carrying the A allele or C allele were co-transfected with pGreen-sh*RXRα* into cells. The mean values (±) were calculated from three independent experiments. Bars, SE. **p < 0.001.

We constructed and transfected a luciferase reporter vector containing the Cx43 gene promoter region with either the A or C allele at rs2071166 into 3 cell lines. Luciferase activity was much lower in the reporter vector containing rs2071166 with the C allele (pGL3-Cx43-C-Luc) than the vector with the A allele (pGL3-Cx43-A-Luc) in the non-RA group (reduced by ~4.53-fold in HEK293, p < 0.001; ~1.90-fold in HeLa, p < 0.001; and ~3.61-fold in HL-1, p < 0.001; Fig. 3a). When RA was added, we observed a significant decrease in relative luciferase activity in cells containing the vector carrying the A allele (Fig. 3b, left), and there was no difference in luciferase activity in cells carrying the vector with the C allele (p = 0.603, HEK293; p = 0.395, HeLa; and p = 0.516, HL-1; Fig. 3b, right).

In our previous research, we transfected pGL3-Cx43-A-Luc (the vector carrying the A allele) into RXRα-knockout cells and found that the relative luciferase activity increased. In this study, we used the same methods to determine rs2071166 function. The results showed that relative luciferase activity increased in cells carrying the A allele vector with shRXRα (increases of ~2.73-fold in HEK293; ~1.71-fold in HeLa; and ~2.82-fold in HL-1; Fig. 3c, left) and that luciferase activity was not changed in cells carrying the C allele vector with shRXRα (p = 0.510, HEK293; p = 0.807, HeLa; and p = 0.122, HL-1; Fig. 3c, right).

### rs2071166 alters RXRα binding to the Cx43 promoter

**Figure 4:**
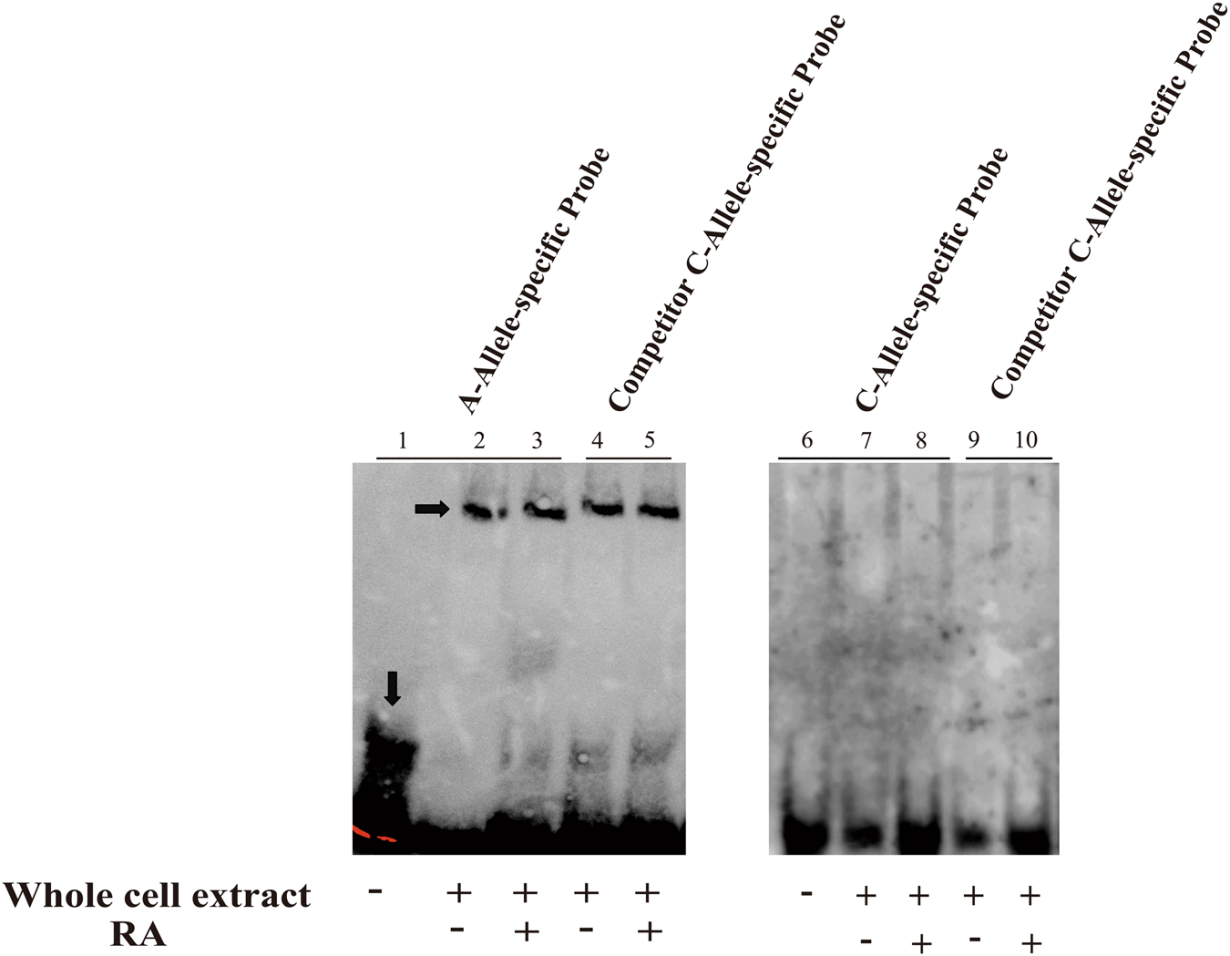
The C allele alters the binding between *RXRα* and the *Cx43* RARE. HeLa whole-cell extracts were treated with DMSO or RA and then incubated with biotin-labeled A allele-specific, C allele-specific and competitor probes in an EMSA. The arrows indicate the free probe and the specific DNA-protein complex shift. Lane 1: A allele-specific probe only; Lanes 2 and 3: A allele-specific probe + protein; Lanes 4 and 5: competitor C allele-specific probe + protein; Lane 6: C allele-specific probe only; Lanes 7 and 8: C allele-specific probe + protein; Lanes 9 and 10: competitor C allele-specific probe + protein.

To confirm the allele-specific interaction of RXRα with the RARE in the Cx43 promoter, we performed an electrophoretic mobility shift assay (EMSA). A binding complex was formed between RXRα and the labeled Cx43 RARE probe with the A allele in the presence or absence of RA. However, no gel shift bands were observed with the C allele probe (Fig. 4).

### The risk factor ‘C’ allele reduces Cx43 promoter activity ***in vivo***

**Figure 5:**
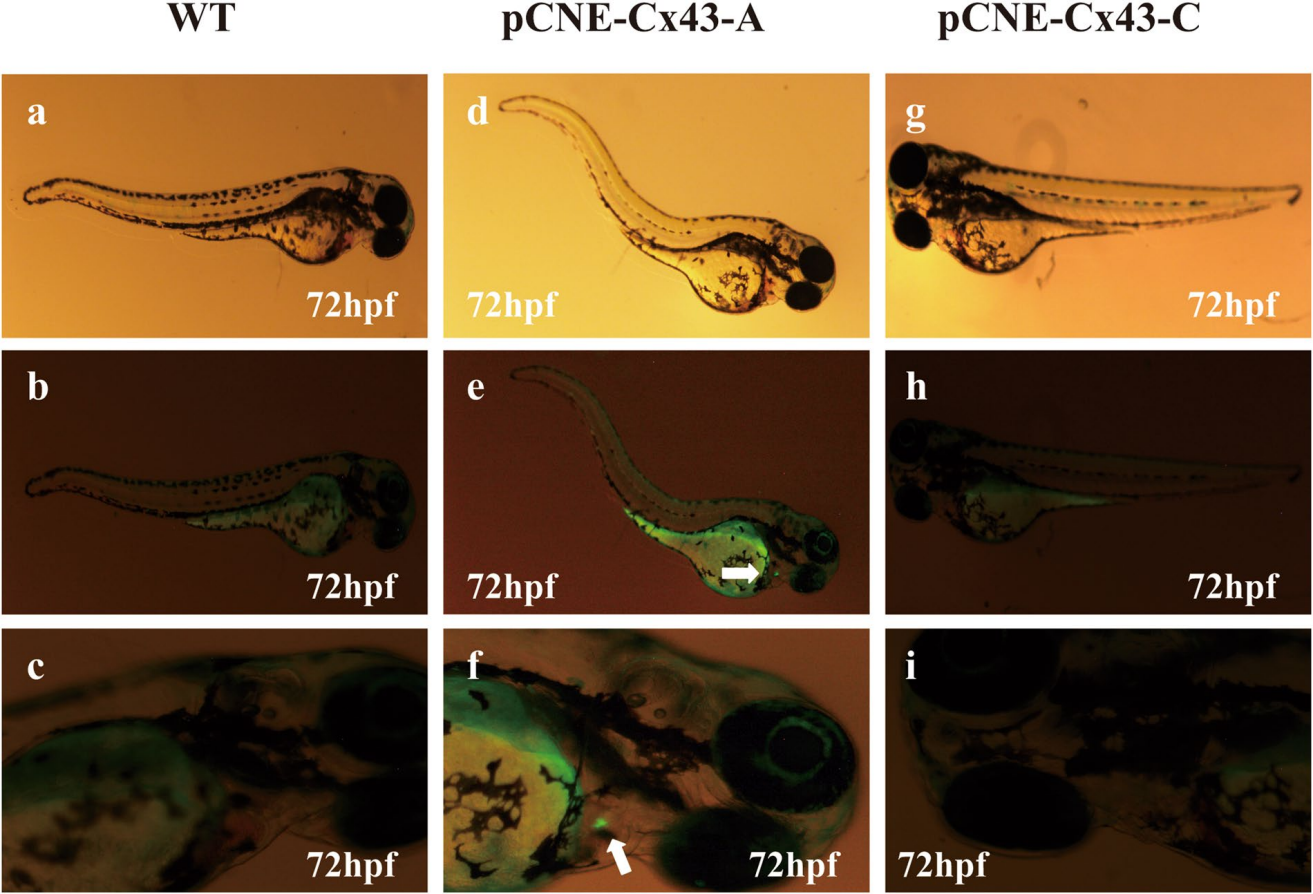
A regulatory variant in Cx43 abrogates RARE function. pCNE-Cx43-A (A allele) or pCNE-Cx43-C (C allele) was injected into zebrafish with Tol2 mRNA. The plasmid containing a 500 bp promoter region within the WT-RARE drove cardiac expression in zebrafish. When mutated, it lost RARE activity. (**a**,**b**,**c**) Wild-type zebrafish without vector injection. (**d**,**e**,**f**) Zebrafish injected with pCNE-Cx43-A. (**g**,**h**,**i**) Zebrafish injected with pCNE-Cx43-C.

We next examined the impact of the variant in zebrafish. We generated a construct carrying a conserved 500 bp region of the Cx43 promoter spanning the variant. We generated constructs with this RARE region harboring each allele of the variant and cloned each into the pCNE-vector. The sequence-verified plasmid was injected with Tol2 mRNA to generate transgenic zebrafish, and fish were observed at 72 h for GFP expression. Of the 154 zebrafish injected with constructs containing the A allele, thirty fish (19.5%) displayed similar expression of GFP in the heart (Fig. 5d,e and f, white arrow). None of the 173 zebrafish injected with the construct containing the C allele expressed GFP in the heart (Fig. 5g,h and i). Together, these results indicate that rs2071166 destroys the activities of gene transfection and RARE function.

## Discussion

Histone acetylation modification plays an important role in transcriptional activation by altering chromatin structure and thus gene expression^19^. Transcription initiation requires RNA-pol II to identify and transcription factors to bind to promoter sequences^24^. When histone acetylation reaches a certain level, nucleosomes loosen, enabling interaction with RNA-Pol II or transcription factors^24^. This process might be exhibit a dose-dependent relationship.

Studies have shown that histone acetylation is associated with gene activation and that H3K18ac is enriched at the promoters of some active genes^25, 26^. Here, we determined that H3K18ac is lower in patients with TOF than in controls and that H3K18ac can bind to the Cx43 promoter directly. We speculate that reduced H3K18ac levels cannot loosen the nucleosomes, resulting in alterations in the expression of Cx43.

CHD are generally caused by both environmental and genetic risk factors. Evidence suggests that single-gene defects can explain only a few CHD cases and that their phenotypes are quite variable^27–29^. Researchers have often hypothesized, but never proven, that most cases of CHD are actually caused by genetic variations and several interacting susceptibility factors^30, 31^.

In recent years, GWAS results have shown that a significant fraction of the potential genetic variation associated with human diseases occurs in regions that are non-protein coding in nature, and experimental investigation of several associated regions has directly implicated regulatory alterations, such as those that disrupt the function of cis-regulatory sequences, influence RNA splicing, and abrogate DNA methylation and miRNA recruitment^32–35^.

Previous studies have identified several SNPs that influence the development of CHD^36–41^. Some investigators believe that SNPs can have dramatic effects on cardiac phenotype, but it is sometimes unlikely that SNPs alone are able to explain the large phenotypic diversity found in different diseases^36^. The functional SNP in methylenetetrahydrofolate reductase (MTHFR) was found to be associated with CHDs such as pulmonary valve stenosis, hypoplastic left heart syndrome, coarctation of the aorta and aortic valve stenosis^37, 38^. Vascular endothelial growth factor (VEGF) polymorphisms are known to be associated with lower VEGF levels *in vitro* and increased risk of TOF and ventricular septal defect (VSD)^39–41^.

However, only a few studies have focused on the association between SNPs in Cx43 and CHDs^2, 42^. In our previous studies^2, 42^, two SNPs in the 3′UTR of Cx43 and two synonymous sequence variants were found in patients with TOF, but there were no differences in the distribution of these SNPs between cases and controls. In the current study, we determined that rs2071166 in Cx43 was associated with an increased risk of TOF. rs2071166 in Cx43 was shown to influence the function of the RARE.

Sequence variations in transcription factor (TF) binding sites may interrupt the binding activity of TFs, leading to abnormal transcriptional expression^21^. Li. *et al*. observed that rs371954924 in WNT5A reduced gene expression by attenuating the binding affinity of the transcription factor SOX9^43^. In our previous study, we identified a functional RARE in the Cx43 promoter^23^. A genetic variant in the Cx43 promoter was located within the functional RARE. We tested its function *in vitro* and *in vivo* in this study. Our research showed that the Cx43 promoter with the minor C allele was activated at a significantly lower level than the Cx43 promoter with the major A allele. The RARE also lost its regulatory function with the C variant. In our previous study, we found that RXRα can bind directly to the RARE^23^. Our EMSA data show that the C allele can alter the interaction between transcription factors and the sequence element. Thus, we believe this SNP can abrogate the binding of RXRα to the RARE.

The injection of a mouse Cx43 promoter-driven GFP construct into zebrafish embryos illustrated some temporospatial expression domains, including the notochord, brain, heart and vasculature^44–46^. For example, GFP-expressing cells appeared in somites at 19 hpf, the notochord at 24 hpf and neural crest cells at 42 hpf. In the present study, we generated a construct containing the human Cx43 promoter with the RARE and injected this into zebrafish embryos, in which we observed GFP expression at 72 h. According to Scott Smemo1^47^, the zebrafish heart has already developed at this stage and begun differentiation and proliferation. These results show that the sequence with the A allele can drive GFP expression in the zebrafish heart at 72 h. In some zebrafish embryos, GFP was also seen in the hematological system and the notochord. These findings are consistent with the results of previous studies^44–46^. However, the construct with the C allele of rs2071166 abrogated GFP expression in zebrafish, and no GFP was expressed in the fish heart. Therefore, these findings might indicate that this low-frequency SNP can destroy the cardiac expression properties of this RARE.

The appropriate distribution of Cx43 maintained the segmental differentiation of the heart tube, the spatial distribution and gradient of tissue-specific morphogenic factors, and segmental differences in the electromechanical activity of the developing tissues^48^. Thus, the production of Cx43 needs to be precisely controlled. It was shown that RVOT pathology is associated with abnormal levels of Cx43 expression^49^. Too much Cx43 could enhance cell differentiation, which might result in an increased ability to regenerate larger and more uniform volumes of tissue^50^.

We went further to examine the Cx43 mRNA level in patients with TOF with different rs2071166 genotypes. It was determined that the Cx43 mRNA level in CC carriers was significantly lower than that in AA/AC carriers. The decreased Cx43 level might result in fewer junctions between cardiomyocytes^51^. Some researchers believe this change could affect the level of intercellular coupling and the local concentration of growth factors (such as PDGF, TGFß, IIGF, and FGF), thus disturbing the coordination of the growth and development of the heart^12^. These findings consistently suggested that the C allele of rs2071166 alters Cx43 function and is therefore associated with TOF.

This is the first study demonstrating the role of altered H3K18ac levels and common Cx43 variants in human patients with sporadic TOF. We determined the level of H3K18ac in patients with TOF and a potential mechanism underlying the regulation of Cx43. In our case-control study described here, the frequencies of CC homozygotes and the C allele were higher in the TOF group than in the healthy control group. Patients with TOF who carried the CC genotype showed lower mRNA levels in RVOT than individuals carrying the AA/AC genotypes. These results are consistent with our functional analysis findings in the luciferase transfection assay and the EMSA. rs2071166 disrupted transcription factor binding and impacted promoter activity.

Studies have shown that SNPs in regulatory and coding regions can modify disease, particularly cancer research studies^52, 53^. SNPs can affect tumor size, anti-tumor drug sensitivity and patient prognosis. Recently, several studies have shown that SNPs might be modifiers in the development of congenital heart disease^54, 55^. It was previously shown that abnormalities in the right ventricular outflow tract are related to an altered level of Cx43 expression^6^. Thus, the identification of this locus might provide new insight into the risk assessment for TOF and contribute to our understanding of the Cx43 molecular mechanisms involved in cardiac neural crest cell migration.

There were some limitations in this study. Our study did not directly prove that rs2071166 in Cx43 is responsible for a higher likelihood of developing TOF. Chromosomal or single gene defects might not be the cause of TOF, but TOF presumably results from genetic variations in several susceptibility factors^39^. In this study, we found that the functional SNP in Cx43 is associated with an increased risk of TOF. Further studies should be completed to determine whether susceptibility to developing TOF is modified by rs2071166 in Cx43.

## Conclusion

In summary, our results indicate that H3K18ac binds to Cx43 and that the acetylation level of H3K18 is reduced in patients with TOF. We speculate that the reduction of H3K18ac in patients with TOF might impact the transcriptional activity and expression level of Cx43. rs2071166 in Cx43 is associated with increased risk of TOF. It also affects the levels of Cx43 mRNA in the patient RVOT and can alter promoter activity and disrupt the interaction between RXRα and RARE.

## Methods

### Patients and tissues for histone acetylation measurement

Heart tissue was obtained from 16 patients with TOF during surgery at the Children’s Hospital of Fudan University in Shanghai, China. The patients receiving surgery also underwent general anesthesia and were on cardiopulmonary bypass (CPB). Thirteen normal heart tissue samples were collected from the Department of Forensic Medicine, Fudan University in Shanghai, China. All the normal tissues were isolated by necrotomy from children who died in traffic accidents or because of other accidental causes. All information is summarized in Table 4. Tissue was stored in formalin immediately after separation from the heart during surgery.

**Table 4:** Basic characteristic of the patients with TOF and controls for IHC.

	Tissue Sample			
	TOF (n = 16)		Control (n = 13)	
	No.	%	No.	%
**Gender**				
Female	8	50.0	6	46.2
Male	8	50.0	7	53.8
Median age (months)	7.5 (3.0–84.0)	48.0 (0.0–201.0)		

The experiments involving these participants were conducted in accordance with the Declaration of Helsinki. The protocols used in this work were reviewed and approved by the Ethics Committee of the Children’s Hospital of Fudan University, Shanghai Medical College of Fudan University prior to the commencement of the study. Informed consent was obtained from the parents or guardians of the children.

### Immunohistochemistry and Measurements

The antibodies used in this study included anti-H3K18ac (1:1000, ab1191, Abcam, Cambridge, UK). For immunostaining, heart tissues were deparaffinized, rehydrated and treated for heat-induced epitope retrieval in sodium citrate (0.01 M, pH 6.0) for 15 min at 70 kPa pressure. As indicated in the manufacturer’s instructions for the SuperPicture 3rd Gen IHC Detection kit (87–9673, Invitrogen, Carlsbad, CA), all sections were treated with peroxidase quenching solution for 5 min at room temperature and incubated in primary antibody at 37 °C for 1 h and then overnight at 4 °C. The next day, the sections were incubated in secondary antibody for 1 h at 37 °C and stained with DAB chromogen. Images were acquired with the Leica DVM2500 digital microscope system (Leica, Wetzlar, Germany). The IHC staining score was assessed with ∑PI (yielding a range from 0 to 12). P represents the percentage of positive cells: a score of 0 points was given for 0% reactivity, 1 point was assigned for 1–10% positive cells, 2 points were assigned for 11–50% positive cells, 3 points were assigned for 51–80% positive cells, and 4 points were assigned for 81–100% positive cells. I represents the intensity and was quantified by the following scores: 0 = negative, 1 = weakly positive, 2 = moderately positive, 3 = strongly positive.

### ChIP- PCR assay

Heart tissues were cross-linked with 1% formaldehyde for 10 min. Immunoprecipitation was performed using a Magnetic ChIP & Enzymatic shearing kit (Active Motif, Carlsbad, CA). Chromatin fragments of 200–400 bp were incubated with 3 µl of rabbit anti-acetyl-histone H3 (Lys18) polyclonal antibody (17–10111, Millipore, Germany) and 5 µl of nonspecific IgG or RNA Pol II provided in the kit at 4 °C overnight. Ten percent of the supernatant volume was reserved as input. The identity and quantity of the DNA fragments were determined via PCR and quantitative real-time PCR. The PCR products were visualized after electrophoresis on a 1.5% agarose gel. Quantitative real-time PCR analysis was completed in accordance with method 1 described in the Magnetic ChIP & Enzymatic shearing kit (Active Motif). The primer pairs that were used for PCR and quantitative real-time PCR are shown in Table 5.

**Table 5:** Primer pairs of the Cx43 for Chromatin immunoprecipitation.

Primer Name	Sequence	Product Size (bp)
Cx43-Fwd	CTGGTTATATGCTTCCCCACCA	122
Cx43-Rev	ACACTGCTTGCTTCACCAGA	
GADPH-Fwd	TACTAGCGGTTTTACGGGCG	166
GADPH-Rev	TCGAACAGGAGGAGCAGAGAGCGA	

### Patient Characteristics and Sample Collection

Patients with TOF were recruited from the Children’s Hospital of Fudan University, Shanghai, China. All patients were diagnosed using an echocardiogram, and the diagnoses were confirmed by surgery. Blood samples were collected from 158 patients with TOF (103 males and 55 females with a median age of 9.0 months) and 300 healthy controls (194 males and 106 females with a median age of 24.0 months) at the Children’s Hospital of Fudan University for DNA sequencing. RVOT tissue samples were collected from 17 patients with TOF undergoing surgical reconstruction. This included nine (52.9%) male and eight (47.1%) female patients with a median age of 8 months (range from 1.5 to 96 months). The tissues were stored in RNAlater Solution (Ambion, Austin, TX) at −80 °C immediately after surgical removal.

The experiments involving these participants were conducted in accordance with the Declaration of Helsinki. The protocols used in this work were reviewed and approved by the Ethics Committee of the Children’s Hospital of Fudan University, Shanghai Medical College of Fudan University prior to the commencement of the study. Informed consent from the parents or guardians of the children was obtained.

### DNA Sequencing

Genomic DNA from 158 patients with TOF (103 males and 55 females with a median age of 9.0 months) and 300 healthy controls (194 males and 106 females with a median age of 24.0 months) was extracted from peripheral blood leukocytes using the QIAamp DNA Blood Mini Kit (QIAGEN, Germantown, MD). The characteristics of the study subjects are summarized in Table 6. The rs2071166 variant site in the Cx43 promoter was amplified by polymerase chain reaction (PCR) with the following primers in all patients and controls (Table 7, Cx43-P1-Fwd and Cx43-P2-Rev). The program of PCR cycles has been described previously^2^. The PCR products were sequenced by Shanghai Invitrogen Co., Ltd. (Shanghai, China). The sequence results were analyzed using Mutation Surveyor demo software (SoftGenetics, Nittany Valley, PA). All variants identified by sequencing were filtered from the National Center for Biotechnology Information human SNP database and the 1000 Genomes Project database^56^.

**Table 6:** Basic characteristic of the patients with TOF and controls for sequencing.

	Blood Sample					
	TOF (n = 158)			Control (n = 300)		
	No.		%	No.		%
**Gende**r						
Female	55		34.8	106		35.3
Male	103		65.2	194		64.7
Median age (months)	9.0 (1.0–144.0)			24.0 (5.0–132.0)		
	**Tissue Sample**					
	**TOF-AA (n = 5)**		**TOF-AC (n = 4)**		**TOF-CC (n = 8)**	
	No.	%	No.	%	No.	%
**Gender**						
Female	3	60	1	25	4	50
Male	2	40	3	75	4	50
Median age (months)	12.0 (5.0–96.0)		7.5 (3.0–14.0)		5.0 (1.5–26.0)

**Table 7:** Primer pairs of the Cx43 for sequence, real-time PCR and plasmid construction.

Primer Name	Sequence	Product Size (bp)
Cx43-P1-Fwd	5′-ATCTCCACCATTCCCTTTGTTA-3′	150
Cx43-P2-Rev	5′-TGGGGAAGCATATAACCAGTTC-3′	
h*Cx43*-Fwd	5′-CAGCTTGTACCCAGGAGGAG-3′	209
h*Cx43*-Rev	5′TGTCCCTGGCCTTGAATATC-3′	
h*GAPDH*-Fwd	5′-CACCAGGTGGTCTCCTCTGAC-3′	150
h*GAPDH*-Rev	5′-GGTGGTCCAGGGGTCTTACTC-3′	
h*B2M*-Fwd	5′-CGCTACTCTCTCTTTCTGG-3′	165
h*B2M*-Rev	5′-TTCTCTCTCCATTCTTCAGTA-3′	
h*Cx43*-inject-Fwd	5′- GATCCTCGAG TAGTTTTCTGGAGACATGGTAGAGG-3′	500
h*Cx43*-inject-Rev	5′-GATCAGATCT TTACACTGCTTGCTTCACCAGA-3′	

### RNA/DNA Isolation and Treatments

Total RNA was isolated from RVOT tissue using Trizol Reagent (Invitrogen, Carlsbad, CA). For each sample, 1 mg of total RNA was used for cDNA synthesis with the PrimeScript RT reagent Kit (Takara Biotechnology, Dalian, China) in a 20 μl reaction mix in accordance with the manufacturer’s instructions. The final cDNA product was diluted 10-fold and subsequently used as a template for real-time PCR.

### Real-time PCR

Amplification reactions were carried out using the SYBR Premix Ex Taq Kit (Takara Biotechnology) and were performed in triplicate in 384-well-plate format using an Applied Biosystems 7900HT Fast Real-time PCR System (Applied Biosystems, Foster City, CA). Relative gene expression levels were calculated using the 2^−ΔΔ^Ct method. Human beta 2 microglobulin (B2M) expression levels were used for the normalization of gene expression values. The primers are listed in Table 7.

### Cell lines and Culture Conditions

The human embryonic kidney cells (HEK293 cells), HeLa cells and HL-1 cells were cultured in Dulbecco’s modified Eagle medium (HyClone, Logan, Utah) that was supplemented with 10% FBS (Gibco BRL, Langley, OK) and penicillin (100 U/ml)-streptomycin (100 µg/ml) and were maintained at 37 °C in a 5% CO_2_ atmosphere.

### Plasmid Construction

#### Reporter Assay

The pGL3-Cx43-A-Luc vector was constructed as described in our previous study^23^. The human Cx43 gene (hCx43) promoter containing rs2071166 was amplified from the DNA of a patient with the CC genotype. Then, these sequences were cloned into pGL3 basic (Promega, Madison, WI) using KpnI and XhoI sites to generate the pGL3-Cx43-C-Luc vectors.

#### Expression Vector

The human pTango-RXRα-Flag and pTango-shRXRα expression plasmids were provided by Chengdu Lingdong Technology Co., Ltd. and have been described in our previous study^23^.

#### Zebrafish Reporter Construction

DNA from a patient with TOF and a healthy control with a homozygous genotype was amplified and cloned into a plasmid containing the e1b minimal fish promoter driving the expression of enhanced GFP (eGFP) protein; this entire region was flanked by tol2 transposon sites. The PCR product was cloned into this vector with the BglII and XhoI sites to produce pCNE-Cx43-A (containing the A allele) and pCNE-Cx43-C (containing the C allele). The primers are listed in Table 7 (hCx43-inject-Fwd and hCx43-inject-Rev).

#### Tol2 Transposase mRNA

Tol2 transposase mRNA was transcribed *in vitro* using the mMessage mMachine Sp6 kit (Ambion).

#### Transfection and Luciferase Assay

The methods used in the transfection can be found in our previous study^23^. Ligand (10 μM RA) was added and incubated for 24 h. Luciferase activity was measured with the dual luciferase assay system (Promega). pRL-Renilla (Promega) was used as a normalization plasmid.

#### Electrophoretic Mobility Shift Assay (EMSA)

We have already described the EMSA protocol in our previous study^23^. Biotin-labeled, double-stranded oligonucleotide probes representing the A allele and C allele RARE sequences were bound to whole-cell extracts (Table 8). The binding reactions were performed using a Light Shift Chemiluminescent EMSA kit (Pierce, Rockford, IL) according to the manufacturer’s protocols.

**Table 8:** EMSA 5′-biotin labeled oligos and unlabeled oligos.

Oligonucleotides Name	Sequence
Biotin-*Cx43*-RARE-Fwd	5′-AATTAGATCATCAAACAGGTCACTCA-3′
Biotin-*Cx43*-RARE-Rev	5′-TGAGTGACCTGTTTGATGATCTAATT-3′
Biotin-allele-specific -Fwd	5′-AATTAGATCATCCAACAGGTCACTCA-3′
Biotin-allele-specific -Rev	5′-TGAGTGACCTGTTGGATGATCTAATT-3′

#### Zebrafish Transgene Assay

pCNE-Cx43-A or pCNE-Cx43-C was coinjected into one- to two-cell stage zebrafish embryos as a 20 ng/μl circular plasmid with 50 ng/μl tol2 transposase mRNA. Zebrafish were maintained using standard protocols^57, 58^, and embryos were obtained from natural crosses of wild-type mating pairs. Embryos were incubated at 28.5 °C, and developing fish were observed daily for promoter activity beginning 24 h after injection. After 72 hours, embryos were stained with methylcellulose solution and examined and photographed with Leica DFC310 FX microscopes (Leica, Wetzlar, Germany).

#### Statistical analysis

Hardy-Weinberg equilibrium was assessed using the Χ^2^ test on the website for the Hardy-Weinberg disequilibrium test (http://ihg.gsf.de/cgi-bin/hw/hwa1.pl). Data were analyzed using SPSS (version 19.0, IBM, Armonk, NY) and GraphPad Prism (version 5.0, GraphPad Software, La Jolla, CA). Differences in allelic or genotypic frequencies between the TOF group and control group were compared using Pearson’s Χ^2^ test. The qRT-PCR data are presented as the mean ± SEM. A Mann-Whitney test of the analysis of variance was conducted on the 2^−ΔΔ^Ct values to compare the mRNA levels between the TOF-CC genotype and TOF-AA genotype groups. Comparisons between the groups in the luciferase transfection assay were made using Student’s t test. All statistical analyses were two sided, and p < 0.05 was considered statistically significant.

## References

[1] Kathiriya IS, Nora EP, Bruneau BG (2015). Investigating the Transcriptional Control of Cardiovascular Development. Circ Res.

[2] Wu Y (2014). Expression of Cx43-related microRNAs in patients with tetralogy of Fallot. World J Pediatr.

[3] Nishii K, Shibata Y, Kobayashi Y (2014). Connexin mutant embryonic stem cells and human diseases. World J Stem Cells.

[4] Salameh A, Blanke K, Daehnert I (2013). Role of connexins in human congenital heart disease: the chicken and egg problem. Front Pharmacol.

[5] Molica F, Meens MJ, Morel S, Kwak BR (2014). Mutations in cardiovascular connexin genes. Biol Cell.

[6] Huang GY (1998). Gap junction-mediated cell-cell communication modulates mouse neural crest migration. J Cell Biol.

[7] Kretz M (2006). Normal embryonic development and cardiac morphogenesis in mice with Wnt1-Cre-mediated deletion of connexin43. Genesis.

[8] Liu S (2006). Distinct cardiac malformations caused by absence of connexin 43 in the neural crest and in the non-crest neural tube. Development.

[9] Reaume AG (1995). Cardiac malformation in neonatal mice lacking connexin43. Science.

[10] Sullivan R (1998). Heart malformations in transgenic mice exhibiting dominant negative inhibition of gap junctional communication in neural crest cells. Dev Biol.

[11] Ward C, Stadt H, Hutson M, Kirby ML (2005). Ablation of the secondary heart field leads to tetralogy of Fallot and pulmonary atresia. Dev Biol.

[12] Kolcz J (2005). The expression of connexin 43 in children with Tetralogy of Fallot. Cellular & molecular biology letters.

[13] Boot MJ, Gittenberger-de Groot AC, Poelmann RE, Gourdie RG (2006). Connexin43 levels are increased in mouse neural crest cells exposed to homocysteine. *Birth defects research*. Part A, Clinical and molecular teratology.

[14] Becanovic K (2015). A SNP in the HTT promoter alters NF-kappaB binding and is a bidirectional genetic modifier of Huntington disease. Nat Neurosci.

[15] Vecellio M (2015). The genetic association of RUNX3 with ankylosing spondylitis can be explained by allele-specific effects on IRF4 recruitment that alter gene expression. Ann Rheum Dis.

[16] Shi J (2017). Alcohol Exposure Causes Overexpression of Heart Development-Related Genes by Affecting the Histone H3 Acetylation via BMP Signaling Pathway in Cardiomyoblast Cells. Alcoholism, clinical and experimental research.

[17] Chang CP, Bruneau BG (2012). Epigenetics and cardiovascular development. Annual review of physiology.

[18] Hung SY, Lin HH, Yeh KT, Chang JG (2014). Histone-modifying genes as biomarkers in hepatocellular carcinoma. International journal of clinical and experimental pathology.

[19] Grunstein M (1997). Histone acetylation in chromatin structure and transcription. Nature.

[20] Struhl K (1998). Histone acetylation and transcriptional regulatory mechanisms. Genes & development.

[21] Huang Q (2015). Genetic study of complex diseases in the post-GWAS era. J Genet Genomics.

[22] Albert FW, Kruglyak L (2015). The role of regulatory variation in complex traits and disease. Nat Rev Genet.

[23] Gu R (2016). Liganded retinoic acid X receptor alpha represses connexin 43 through a potential retinoic acid response element in the promoter region. Pediatric research.

[24] Zhong L (2010). Ethanol and its metabolites induce histone lysine 9 acetylation and an alteration of the expression of heart development-related genes in cardiac progenitor cells. Cardiovascular toxicology.

[25] Shahbazian MD, Grunstein M (2007). Functions of site-specific histone acetylation and deacetylation. Annual review of biochemistry.

[26] Wang Z (2008). Combinatorial patterns of histone acetylations and methylations in the human genome. Nature genetics.

[27] Garg V (2003). GATA4 mutations cause human congenital heart defects and reveal an interaction with TBX5. Nature.

[28] Goldmuntz E, Geiger E, Benson DW (2001). NKX2.5 mutations in patients with tetralogy of fallot. Circulation.

[29] Yagi H (2003). Role of TBX1 in human del22q11.2 syndrome. Lancet.

[30] Brennan P, Young ID (2001). Congenital heart malformations: aetiology and associations. Seminars in neonatology: SN.

[31] Baldini A (2002). DiGeorge syndrome: the use of model organisms to dissect complex genetics. Human molecular genetics.

[32] Sakabe NJ, Savic D, Nobrega MA (2012). Transcriptional enhancers in development and disease. Genome biology.

[33] Harismendy O (2011). 9p21 DNA variants associated with coronary artery disease impair interferon-gamma signalling response. Nature.

[34] Pittman AM (2009). The colorectal cancer risk at 18q21 is caused by a novel variant altering SMAD7 expression. Genome research.

[35] Wasserman NF, Aneas I, Nobrega MA (2010). An 8q24 gene desert variant associated with prostate cancer risk confers differential *in vivo* activity to a MYC enhancer. Genome research.

[36] Muntean I, Toganel R, Benedek T (2017). Genetics of Congenital Heart Disease: Past and Present. Biochemical genetics.

[37] Junker R (2001). Infant methylenetetrahydrofolate reductase 677TT genotype is a risk factor for congenital heart disease. Cardiovascular research.

[38] Wang W (2013). Association between 5, 10-methylenetetrahydrofolate reductase (MTHFR) polymorphisms and congenital heart disease: A meta-analysis. Meta gene.

[39] Lambrechts D (2005). Low expression VEGF haplotype increases the risk for tetralogy of Fallot: a family based association study. Journal of medical genetics.

[40] Li X (2015). VEGF Gene Polymorphisms are Associated with Risk of Tetralogy of Fallot. Medical science monitor: international medical journal of experimental and clinical research.

[41] Xie J (2007). VEGF C-634G polymorphism is associated with protection from isolated ventricular septal defect: case-control and TDT studies. European journal of human genetics: EJHG.

[42] Huang GY (2011). Evaluating the role of connexin43 in congenital heart disease: Screening for mutations in patients with outflow tract anomalies and the analysis of knock-in mouse models. J Cardiovasc Dis Res.

[43] Li P (2015). Variants in the Regulatory Region of WNT5A Reduced Risk of Cardiac Conotruncal Malformations in the Chinese Population. Scientific reports.

[44] Jiang Q (2010). Critical role of connexin43 in zebrafish late primitive and definitive hematopoiesis. Fish physiology and biochemistry.

[45] Chatterjee B (2001). Analysis of Cx43alpha1 promoter function in the developing zebrafish embryo. Cell communication & adhesion.

[46] Chatterjee B (2005). Developmental regulation and expression of the zebrafish connexin43 gene. Developmental dynamics: an official publication of the American Association of Anatomists.

[47] Smemo S (2012). Regulatory variation in a TBX5 enhancer leads to isolated congenital heart disease. Human molecular genetics.

[48] Guthrie SC, Gilula NB (1989). Gap junctional communication and development. Trends in neurosciences.

[49] Huang GY (1998). Alteration in connexin 43 gap junction gene dosage impairs conotruncal heart development. Dev Biol.

[50] Rossello RA, Wang Z, Kizana E, Krebsbach PH, Kohn DH (2009). Connexin 43 as a signaling platform for increasing the volume and spatial distribution of regenerated tissue. Proceedings of the National Academy of Sciences of the United States of America.

[51] Saffitz JE, Green KG, Kraft WJ, Schechtman KB, Yamada KA (2000). Effects of diminished expression of connexin43 on gap junction number and size in ventricular myocardium. American journal of physiology. Heart and circulatory physiology.

[52] Eun YG (2015). Single nucleotide polymorphisms of the Fas gene are associated with papillary thyroid cancer. Auris, nasus, larynx.

[53] Bhowmik A, Nath S, Das S, Ghosh SK, Choudhury Y (2015). ATM rs189037 (G > A) polymorphism and risk of lung cancer and head and neck cancer: A meta-analysis. Meta gene.

[54] Li XT (2015). Association of TGFBR2 rs6785358 Polymorphism with Increased Risk of Congenital Ventricular Septal Defect in a Chinese Population. Pediatr Cardiol.

[55] Zhang J (2015). Association of GDF1 rs4808863 with fetal congenital heart defects: a case-control study. BMJ open.

[56] http://www.internationalgenome.org/1000-genomes-browsers.

[57] Kimmel CB, Ballard WW, Kimmel SR, Ullmann B, Schilling TF (1995). Stages of embryonic development of the zebrafish. Developmental dynamics: an official publication of the American Association of Anatomists.

[58] Whitlock KE, Westerfield M (2000). The olfactory placodes of the zebrafish form by convergence of cellular fields at the edge of the neural plate. Development.

